# Utility of biomarkers and temporal artery biopsy length for investigating giant cell arteritis in Western Australia

**DOI:** 10.1111/1756-185X.14488

**Published:** 2022-11-19

**Authors:** Isabella S. Atlas, Stephen M. Colley, Mark A. Chia

**Affiliations:** ^1^ Sir Charles Gairdner Hospital Perth Australia; ^2^ Fremantle Hospital Perth Australia; ^3^ Institute of Ophthalmology University College London London UK

**Keywords:** biopsy, C‐reactive protein, epidemiology, erythrocyte sedimentation rate, giant cell arteritis, inflammatory markers

## Abstract

**Aim:**

To explore demographic characteristics, biopsy length, and blood biomarker performance in an Australian cohort of patients who have undergone temporal artery biopsy (TAB) for giant cell arteritis (GCA).

**Methods:**

We extracted data on biopsies performed for GCA between January 2016 and December 2020 at public hospitals in Perth. Sensitivity, specificity, and area under the curve (AUC) were calculated for blood results. We evaluated the proportion of biopsies with post‐fixation length less than 15 mm and explored several length associations.

**Results:**

We retrospectively reviewed biopsies of 360 patients (65.8% female, mean age 72.1 years). Biopsy‐positive patients were older (6.0 years, *P* < 0.01), and had higher C‐reactive protein (CRP) (44.5 mg/L, *P* < 0.01), erythrocyte sedimentation rate (ESR) (18.9 mm/h, *P* < 0.01), and platelets (86.8 × 10^3^/μL, *P* < 0.01) compared with biopsy‐negative patients. CRP and platelets had the highest AUCs at 0.76 and 0.71, respectively. Sensitivities for CRP and ESR were 96.2% and 91.5%, respectively. Specificities were comparatively low at 41.3% for CRP and 37.4% for ESR. The proportion of biopsies with sub‐optimal length was 55.9% and this varied significantly by site (*P* < 0.01). Smaller sites performed worse, with a sub‐optimal biopsy rate of 87% amongst the three smallest sites.

**Conclusion:**

ESR and CRP are helpful preliminary investigations, especially in identifying low‐risk patients, but their specificity is limited. Smaller centers had a higher proportion of biopsies with sub‐optimal length. Considering the importance of biopsy length for TAB diagnostic value, reviewing biopsy data may assist services in developing improvement strategies.

## 1 INTRODUCTION

Giant cell arteritis (GCA), also known as temporal arteritis, is a systemic vasculitis of medium and large vessels that predominantly affects branches of the carotid and vertebral arteries.^1^ It is the most common systemic vasculitis affecting people over 50 years.^1^ Clinical manifestations include temporal headache, scalp tenderness, jaw claudication, and low‐grade fever.^1^ With involvement of the ophthalmic artery, GCA can cause an ischemic optic neuropathy, which can rapidly progress to the contralateral eye leading to severe, permanent, bilateral visual loss.^1^, ^2^ The diagnosis and treatment of GCA is therefore an ophthalmologic emergency.

Temporal artery biopsy (TAB) is the gold standard for diagnosis but is an invasive procedure requiring specialist involvement. As a result, clinicians often use biomarkers in conjunction with demographic and clinical features to help guide their decision‐making. Commonly used biomarkers include erythrocyte sedimentation rate (ESR), C‐reactive protein (CRP), platelets, and hemoglobin. Previous work has assessed the utility of these biomarkers in predicting biopsy‐positive GCA,^3^, ^4^, ^5^ but to our knowledge, this has not been evaluated within an Australian population. The incidence of GCA is known to vary among different ethnicities.^6^ This type of variability can impact diagnostic performance, therefore the best approach is for clinicians to base their decisions on studies that most closely match their personal clinical context. This underpins the importance of evaluating biomarker performance in a broad range of populations.

Histopathologic features of GCA include a necrotizing arteritis, characterized by a predominance of mononuclear cell infiltrates or a granulomatous process with multinucleated giant cells.^7^ As a result of the occurrence of skip lesions, small biopsy samples may fail to capture pathological areas, leading to false‐negative results.^8^ Several studies have investigated the optimal length required to maximize diagnostic yield, with a recent paper concluding that clinicians should aim for a pre‐fixation length of 15‐20 mm.^9^, ^10^ Sub‐optimal biopsies may lead to inadequate treatment or the need for additional procedures, therefore TAB length is an important outcome measure.

This study attempts to improve the diagnosis of GCA by pursuing three major aims. First, we describe the demographic and biochemical characteristics of a multi‐center cohort of patients who have undergone TAB for GCA and compare this with international TAB cohorts. Second, we evaluate the predictive value of blood results for detecting biopsy‐positive GCA, specifically within an Australian population. Finally, we audit biopsy length in this cohort in accordance with recent recommendations.

## 2 MATERIALS AND METHODS

We performed a retrospective, multi‐center, cross‐sectional study of patients who underwent TAB for GCA. Ethics approval was granted through a local institutional procedure for clinical audits (Western Australian Health Governance Evidence Knowledge Outcomes, Approval: 39765) and the study was conducted in accordance with the Declaration of Helsinki. Individual patient consent was not required because of the retrospective nature of the study.

### 2.1 Study population

We reviewed patients who underwent TAB for GCA between January 2016 and December 2020 at seven public hospitals in Perth, Western Australia. The involved sites incorporated all Perth tertiary hospitals and all tertiary eye services, representing approximately 80% of public adult ophthalmology admissions across the metropolitan area.^11^

### 2.2 Data extraction

We used hospital procedural codes to identify patients who had undergone TAB for GCA and extracted demographic data, blood results, and biopsy details using a digital pathology platform (iSoft Clinical Manager). Comprehensive information on clinical features and treatment was not available.

To evaluate the utility of biomarkers, we used the highest value for ESR, CRP, and platelets, and the lowest value for hemoglobin, within 1 month before the date of biopsy. This decision was made in an attempt to capture treatment‐naive blood results, in the absence of a method to determine the exact timing of steroids. A positive biopsy, as identified by the reporting pathologist, was the sole criterion for the diagnosis of biopsy‐positive GCA.

### 2.3 Statistical analysis

We performed statistical analysis using SPSS Statistics Version 26 (IBM Corporation, Armonk, NY, USA).^12^ Pearson's χ^2^ tests and independent sample *t* tests were used to evaluate differences between biopsy‐positive and biopsy‐negative groups for categorical and continuous variables, respectively. To calculate measures of diagnostic performance, we categorized blood results as binary outcomes using age and sex‐specific reference ranges as detailed in Table S1. We calculated the proportion of biopsies with optimal length based on a minimum post‐fixation cut‐off of 15 mm^10^ and explored several biopsy‐length associations.

## 3 RESULTS

### 3.1 Participants

Between January 2016 and December 2020, data were explored for a total of 360 patients (65.8% female, mean age 72.1 years) investigated for GCA. Eighty‐six biopsies (23.9%) were positive for GCA and 19 (5.3%) showed indeterminate histopathologic findings. Table 1 summarizes the demographic factors and biomarker results across biopsy‐positive and biopsy‐negative patients.

**TABLE 1:** Comparison of demographic factors and biomarker results between biopsy‐positive and biopsy‐negative patients

	Biopsy‐positive *n* = 86	Biopsy‐negative *n* = 255	*P* value
Mean age (SD), years	76.7 (8.4)	70.6 (11.1)	<0.001*
Sex			0.90
Male	30 (35%)	87 (34%)	
Female	56 (65%)	168 (66%)	
Indigenous	0 (0%)	5 (2.0%)	0.19
Mean biomarker result (SD)			
ESR (mm/h)	68.9 (25.7)	50.1 (32.4)	<0.001*
CRP (mg/L)	88.5 (80.2)	44.0 (75.8)	<0.001*
Platelets (×10^3^/μL)	409.7 (119.7)	322.9 (127.8)	<0.001*
Hemoglobin (g/L)	118.0 (15.6)	121.3 (20.3)	0.15
Mean biopsy length (SD), mm	15.5 (8.7)	14.3 (7.3)	0.21
Length adequacy			0.29
Optimal	41 (47.7%)	104 (41.1%)	
Sub‐optimal	45 (52.3%)	149 (58.9%)	

Abbreviations: CRP, C‐reactive protein; ESR, Erythrocyte sedimentation rate; SD, standard deviation.

* Statistically significant (*P* < 0.05).

The mean age at time of diagnosis was 76.7 ± 8.4 years. Compared with the biopsy‐negative group with a mean age of 70.6 ± 11.1 years, biopsy‐positive patients were significantly older (*P* < 0.001). Sex proportions were not significantly different between groups. Over the 5 year period, only five Aboriginal and Torres Strait Islander patients underwent TAB, all of whom were female and had negative biopsy results.

Biopsy‐positive patients had significantly higher CRP (44.5 mg/L, 95% confidence interval [CI] 24.3‐64.7, *P* < 0.001), ESR (18.9 mm/h, 95% CI 11.7‐26.0, *P* < 0.001), and platelets (86.7 × 10^3^/μL, 95% CI 53.9 × 10^3^‐119.7 × 10^3^, *P* < 0.001). The difference in hemoglobin between biopsy‐negative and biopsy‐positive patients was not significant (*P* = 0.15).

### 3.2 Predictive value of blood results

The sensitivity, specificity, positive predictive value (PPV), and negative predictive value (NPV) for biomarkers are reported in Table 2. The biomarkers with the best overall discriminating ability in our study were CRP and platelet count with areas under the curve (AUCs) of 0.76 and 0.71, respectively. CRP was the most sensitive biomarker (96.2%), but platelet count was the most specific (79.3%). When considering ESR and CRP as composite outcomes (both elevated or either elevated), there were trade‐offs between sensitivity and specificity compared with interpreting biomarkers individually (Table 2). Hemoglobin had poor discriminating ability in our study (AUC 0.44).

**TABLE 2:** Measures of diagnostic performance including sensitivity, specificity, predictive value, and AUC of serum biomarkers (mean and range)

	Sensitivity, %	Specificity, %	PPV, %	NPV, %	AUC
ESR	91.5 (84.1, 96.2)	37.4 (31.1, 44.1)	36.2 (29.9, 42.9)	91.9 (84.9, 96.4)	0.68 (0.62, 0.74)
CRP	96.2 (90.3, 99.0)	41.3 (34.7, 48.1)	38.3 (31.6, 45.2)	96.6 (91.4, 99.1)	0.76 (0.71, 0.82)
ESR and CRP	91.0 (83.4, 96.0)	51.2 (44.4, 58.1)	42.0 (34.7, 49.5)	93.6 (88.1, 97.2)	*
ESR or CRP	96.2 (90.3, 99.0)	27.4 (21.5, 33.8)	33.9 (27.9, 40.3)	94.8 (87.1, 98.7)	*
Hemoglobin	61.7 (50.9, 71.8)	52.2 (45.7, 58.7)	31.8 (24.9, 39.4)	79.1 (72.0, 85.1)	0.44 (0.37, 0.51)
Platelets	51.9 (40.9, 62.9)	79.3 (73.6, 84.4)	47.6 (37.1, 58.3)	82.0 (76.4, 86.9)	0.71 (0.65, 0.78)

Abbreviations: AUC, area under the curve; CRP, C‐reactive protein; ESR, erythrocyte sedimentation rate; PPV, positive predictive value; NPV, negative predictive value.

Values in brackets are 95% confidence intervals.

* AUC cannot be calculated for composite outcomes because of the involvement of multiple thresholds.

### 3.3 Biopsy length

**FIGURE 1:**
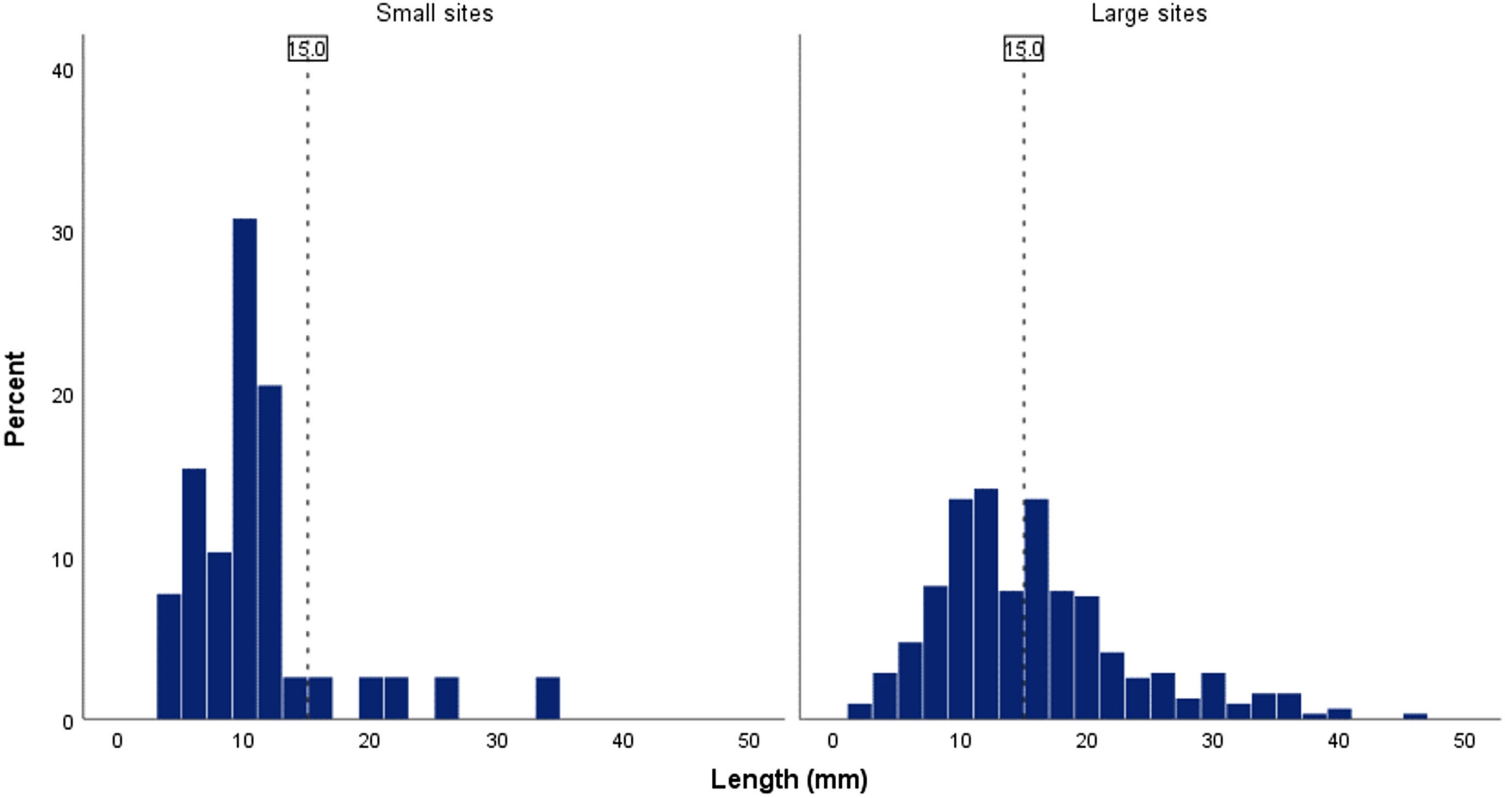
Biopsy length distribution at the three hospital sites performing fewer than 30 temporal artery biopsies over 5 years (left) compared with the four sites performing more than 30 over 5 years (right). Vertical line represents optimal biopsy length of at least 15 mm

The mean biopsy length was 15.49 ± 8.75 mm for the biopsy‐positive patients and 14.29 ± 7.29 mm for the biopsy‐negative patients. There was no significant difference in biopsy length between the groups (*P* = 0.29). When considering only patients with an elevated CRP, there was still no difference in biopsy length (*P* = 0.17) between biopsy‐positive (16.05 ± 8.64 mm) and biopsy‐negative (14.45 ± 7.49 mm) groups. Of the TABs performed in Perth during the study period, 55.9% were below the optimal diagnostic length. The proportion of sub‐optimal biopsies varied significantly by site (Fisher's exact test *P* < 0.01); this is both detailed in Table 3 and depicted in Figure 1.

**TABLE 3:** Proportions of sub‐optimal (<15 mm) and optimal (≥15 mm) temporal artery biopsies by hospital site

	Site 1	Site 2	Site 3	Site 4	Site 5	Site 6	Site 7
Total number of biopsies	6	11	22	46	74	74	125
Sub‐optimal biopsy length							
Number of biopsies	6	9	19	28	44	40	54
Percentage of site total	100%	81.8%	86.4%	60.9%	59.5%	54.1%	43.2%
Optimal biopsy length							
Number of biopsies	0	2	3	18	30	34	71
Percentage of site total	0%	18.2%	13.6%	39.1%	40.5%	45.9%	56.8%

## 4 DISCUSSION

In our study investigating the utility of biomarkers in the workup for GCA before TAB, we found that biopsy‐positive patients had significantly higher CRP, ESR, and platelet count. CRP had the highest sensitivity, platelets the greatest specificity, and hemoglobin was not a useful measure. Notably, over 50% of the 360 TABs performed were below the optimal post‐fixation length of 15 mm. This finding prompts further consideration of the importance of TAB length as we discuss the implications of suboptimal specimens, acknowledging the limitations of our study in drawing impactful conclusions alone. These results emphasize the potential value in a larger‐scale study in this area.

To our knowledge, this is the third study describing a hospital TAB cohort in an Australian population. The mean age at time of diagnosis was 76.7 ± 8.4 years. This is comparable to the findings of De Smit et al. and Dunstan et al. in their Australian studies, who found a mean age of 76.4 and 78 years, respectively.^13^, ^14^ This result is also similar to the findings of several international studies.^3^, ^4^, ^15^, ^16^ Sixty‐five percent of biopsy‐positive patients were female, similar to the 68% within the South Australian cohort investigated by Dunstan et al.^13^ and 66% in a large international meta‐analysis.^17^ We found no significant difference in the rate of positive biopsy results between female and male patients. This can be attributed to the higher proportion of women undergoing TAB, potentially due to clinical features of GCA, particularly headache, occurring more frequently in women,^18^ as well as higher clinical suspicion of diagnosis in female patients given the known predilection. There were five Indigenous patients in our cohort, all of whom had negative TAB results. In comparison with non‐Indigenous Australians, it has been observed that Indigenous Australians have increased frequencies of certain autoimmune diseases, such as rheumatic fever and post‐streptococcal glomerulonephritis, but decreased frequency of many others, such as rheumatoid arthritis and celiac disease.^19^ Further study of GCA in Indigenous Australians is required to explore this relationship.

CRP was found to be the single most useful biomarker in our cohort for the workup for GCA before TAB with a sensitivity of 96.2% and a specificity of 41.3%. This result is congruent with the findings of several other studies.^4^, ^5^, ^17^ CRP has a more rapid response to inflammatory stimuli than ESR,^20^ which contributes to its utility given the acute symptomatology of GCA. Additionally, CRP is not influenced by age to the same degree as ESR, which may contribute to its reliability.^21^ In our study cohort, the sensitivity of ESR was comparable to previously reported results (78%‐93%).^17^ Hemoglobin performed poorly with a sensitivity of 61.7% and specificity of 52.2%. We did not consider whether the anemia was explained by another cause in this study, as specified in the American College of Rheumatology 1990 criteria,^7^ because data could not be accurately obtained. This consideration may have increased its diagnostic performance.

Among the 86 patients with positive TAB, three had negative inflammatory markers, representing 3.5% of biopsy‐positive patients. A recent literature review of retrospective studies found that the presence of negative inflammatory markers in patients diagnosed with GCA varied from 1.4% to 22.5%.^22^ This study found that among these patients, there was a predominance of female individuals, with headache and visual symptoms the most common symptoms reported.^22^

In all, 55.9% of TABs performed in Perth during the study period were below the optimal length of 15 mm. If our approach to auditing biopsy length was widely adopted by other health services and subsequent steps were made to improve performance in this area, this could have a significant impact on the sensitivity of TAB. Increasing its sensitivity would influence topical decision‐making around the recommendations for the gold standard investigation for GCA. We encourage other health centers internationally to review their current compliance with evidence‐based TAB length recommendations. A 2016 study in Sydney, Australia found that there had already been a significant increase in length of the TABs performed from 2008 to 2014 compared with between 2000 and 2005.^23^ We also found that adequacy of biopsy length was affected by the hospital site at which the test was performed. Smaller‐capacity hospital sites, undertaking fewer biopsies appeared to perform worse, with a combined sub‐optimal biopsy rate of 87% for the three smallest sites. Possible factors that may explain this discrepancy include: (a) greater procedural experience in surgeons at sites with a higher surgical volume, (b) a tendency for supervised teaching facilities to more rapidly adopt recent guidelines, (c) variations in specialty distribution (vascular surgery, general surgery, ophthalmology) and training level of surgeons performing procedures, and (d) a tendency towards risk minimization in less intensively resourced facilities. Health services should consider this evidence and review biopsy data to assist in developing strategies for improvement. When targeting optimal biopsy length, surgeons must also consider shrinkage after specimen fixation, with a reported mean shrinkage of 2.4 mm.^24^

Our study has relevant limitations. First, because of our inability to access comprehensive medical records across all sites, we were not able to consider clinical features in our analysis. The definition of GCA was therefore limited to biopsy‐positive GCA rather than incorporating clinical criteria as defined by the American College of Rheumatology 1990. Second, we were not able to account for the potential impact of steroid treatment on blood and biopsy results because information on the timing of treatment was limited. We attempted to minimize this impact by selecting a pre‐biopsy window of 1 month for blood results, which likely captured a treatment‐naive result. Similarly, we anticipate the impact on biopsy results to be small, as histopathologic evidence is typically maintained for at least a month after therapy.^25^, ^26^ Third, our estimations of the predictive value of blood results are sub‐optimal because our retrospective cohort was defined according to the presence of a reference test (biopsy). This would exclude patients who had bloods performed as part of a GCA workup but who did not undergo biopsy. As a result of the invasive nature of the procedure, it would likely not be feasible to perform an ideal diagnostic accuracy study in which all patients considered for GCA undergo biopsy, therefore this more limited approach is commonly used.^3^, ^4^, ^27^, ^28^

## 5 CONCLUSION

The demographic and biochemical profile of our Western Australian TAB cohort was similar to other Western populations. ESR and CRP were found to be helpful preliminary investigations in the workup for GCA, especially in identifying low‐risk patients, but their specificity is limited. Over 50% of TABs performed were below the optimal length. Adequacy of biopsy length was affected by testing site, with larger centers performing a higher proportion of biopsies with optimal length. Considering the importance of biopsy length for TAB diagnostic value, reviewing biopsy data may assist health services in developing strategies for improvement. GCA is a devastating disease to miss and given Australia's aging population and evolving diagnostic methods, GCA should remain a research focus.

## AUTHOR CONTRIBUTIONS

Conception and design: SC and MC. Data collection: IA and MC. Statistical analysis: MC. Manuscript drafting: IA. All authors contributed to the critical revision of the manuscript for important intellectual content, approved the final version and are accountable for the integrity of its content.

## FUNDING INFORMATION

None.

## CONFLICT OF INTEREST

The authors declare that there are no conflicts of interest.

## Supporting information


**Table S1** Biomarker cut‐offs by gender
