# Supplementary material for: Utility of biomarkers and temporal artery biopsy length for investigating giant cell arteritis in Western Australia
Source: Int J Rheum Dis. 2022 Nov 19;26(2):286–91. doi: 10.1111/1756-185X.14488 (PMC10098702; doi:10.1111/1756-185X.14488)
Supplement: Supplementary file 1 — Table S1 Biomarker cut‐offs by gender [file APL-26-286-s001.docx]

| Table S1. Biomarker cut-offs by gender | | |  |
| --- | --- | --- | --- |
| Biomarker | Abnormal result in males | Abnormal result in females | Source |
| ESR | >30mm/h | >35mm/h | PathWest |
| CRP | >8mg/L | >8mg/L | Kermani et al^4^ |
| Haemoglobin | <135g/L | <115g/L | PathWest |
| Platelet count | >400x10^3^/μL | >400x10^3^/μL | PathWest |
| CRP = C-reactive protein, ESR = Erythrocyte sedimentation rate | | |  |
